# Classification of vocalizations by recordings from the auditory midbrain

**DOI:** 10.1186/1471-2202-13-S1-P89

**Published:** 2012-07-16

**Authors:** Dominika Lyzwa, Michael Herrmann

**Affiliations:** 1 Dept. of Nonlinear Dynamics, Max Planck Inst. for Dynamics and Self-Organization, Göttingen, 37077, Germany; 2 Institute of Perception, Action and Behavior, University of Edinburgh, Edinburgh, EH8 9AB, UK

## 

The temporal and spatial properties of responses to complex stimuli in the central nucleus of the inferior colliculus (ICC), the main converging station in the auditory midbrain, can provide evidence for coding principles in the auditory system and are relevant for the design of neuroprosthesis.

We study responses from guinea pigs to a set of eleven species-specific vocalizations which show a wide range of spectral contents, envelope types, frequency and amplitude modulations. The envelopes of the acoustically presented stimuli are characterized as complex or periodic impulses and have various degrees of periodicity. The frequency content ranges from harmonic strucutres to broad spectral distributions. The data analyzed were multi-unit recordings taken simultaneously from 32 positions in the ICC of guinea pigs using a double shank electrode. Peristimulus time histograms (PSTHs) of the high dimensional recordings were classified by linear discriminant analysis in order to evaluate the spatial and temporal distribution of stimulus-related information without the assumption of a specific coding scheme. Neighboring neural populations respond in a similar manner and have highly correlated PSTHs. Combining responses from different positions improves the classification performance for distant postions which do not show correlation, but not for adjacent positions.

**Figure 1:**
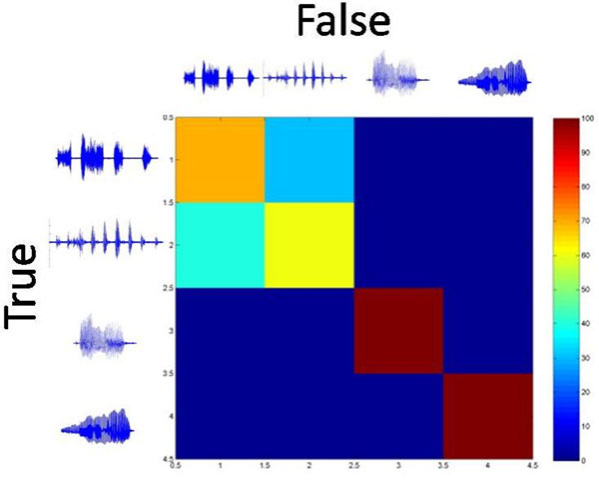
Confusion matrix for the classification of neural responses to periodic low-frequency stimuli (low chutter, drr) and complex-envelope broad-spectrum stimuli (squeal, low whistle) for a multi-unit in an iso-frequency lamina with a characteristic frequency of approx. 20kHz.

Separability of responses along the tonotopic gradient of the ICC to vocalizations shows great variation according to their spectral properties. Low-frequency stimuli are found to be better separable in low characteristic frequency (CF) lamina than complex envelope or broad spectral stimuli. Some stimuli of the latter type (squeal, whistle) are nearly perfectly discriminated in multi-units with high CF whereas discrimination for low-frequency stimuli is less good (60 - 70 %) in this region, see Figure 1. For multi-units positioned in the mid-frequency range along the tonotopic axis all stimuli can be separated at a level similar to the result obtained in the low/high CF regions for the respective preferred stimuli.

We interpret the results as a consequence of the combination of several frequencies in the vocalizations that are better covered by the widely tuned receptive fields of the neurons in the mid-frequency range than by the receptive fields of the units with CFs in the extreme frequency ranges.

